# Direct formation of carbon nanotube wiring with controlled electrical resistance on plastic films

**DOI:** 10.1038/s41598-023-29578-w

**Published:** 2023-02-08

**Authors:** Hiroaki Komatsu, Takahiro Matsunami, Yosuke Sugita, Takashi Ikuno

**Affiliations:** Department of Applied Electronics, Graduate School of Advanced Engineering, Tokyo University of Science, Katsushika, Tokyo, 125-8585 Japan

**Keywords:** Carbon nanotubes and fullerenes, Electrical and electronic engineering

## Abstract

We have developed a simple method to fabricate multi-walled carbon nanotube (MWNT) wiring on a plastic film at room temperature under atmosphere pressure. By irradiating a MWNT thin film coated on a polypropylene (PP) film with a laser, a conductive wiring made of a composite of MWNT and PP can be directly fabricated on the PP film. The resistance of MWNT wiring fabricated using this method were ranging from 0.789 to 114 kΩ/cm. By changing the scanning speed of laser, we could fabricate various regions with different resistances per unit length even within a single wiring. The formation mechanism of the MWNT wiring with tunable resistance was discussed from both experimental results, such as microscopic structural observation using cross-sectional scanning electron microscopy and microscopic Raman imaging, and simulation results, such as heat conduction in the film during local laser heating. The results suggest that the MWNT wiring was formed by PP diffusion in MWNT at high temperature. We also demonstrated that excess MWNTs that were not used for wiring could be recovered and used to fabricate new wirings. This method could be utilized to realize all-carbon devices such as light-weight flexible sensors, energy conversion devices, and energy storage devices.

## Introduction

Flexible all-carbon devices have attracted attention as post-silicon devices owing to their flexibility, light weight, and excellent physical and chemical properties^1–3^. Carbon nanotube (CNT) is one of the most promising building blocks for flexible all-carbon devices because of its intriguing physical and chemical properties^4^. Recently, in addition to CNT devices on rigid substrates^5,6^, CNT devices on flexible substrates such as plastic films have been widely reported^7–11^. CNT-based flexible devices are generally fabricated by the following steps because typical flexible substrates are unavailable for the high-temperature growth process^12^. First, CNTs are grown on rigid substrates by chemical vapor deposition (CVD). Then, CNTs are patterned by lithography processes. Finally, the CNT wirings are transferred onto a flexible substrate^13^. This method has two issues: one of them is that sequential processes including the high-temperature process and the clean room process are required. The other is that since the electrical resistance of the transferred CNT wiring is determined by the resistance of the CNT films before the transfer, to produce CNT wiring of various resistance values, repeated transfer processes are required. Therefore, it is necessary to develop a simple process that can form CNT wirings with controlled resistance directly on plastic substrates.

Two primary methods for fabricating CNT wirings on plastic substrates directly, so-called the laser-induced forward transfer (LIFT) method^14^ and the thermal fusion (TF) method^15–18^, have been reported. The LIFT method is a technology in which a material irradiated by a laser is transferred to a target substrate in proximity, thus achieving direct-write of CNT wiring independent of substrate materials^19^. LIFT methods can transfer CNTs to various substrates such as aluminum, polyimide, glass, and quartz by laser irradiation through patterned masks^20–22^. In the LIFT method, controlling the resistance of CNT wirings is difficult because it is necessary to prepare donor material with different resistance. In addition, the LIFT method usually requires expensive pulsed lasers. In the TF methods, CNTs were mixed with polymers including polypropylene (PP), polycarbonate (PC), and epoxy in advance^15–18^. The composite was then locally heated using a laser to vaporize the polymers selectively. As a result, CNT wiring was formed. The TF method can control the resistance of CNT wiring on a flexible substrate by changing the laser conditions. For example, the resistance of the CNT wiring was reported to range from 0.021 to 464 kΩ/cm as the laser conditions varied^17^. However, the TF method is problematic because CNTs must be mixed with the polymer in advance, and for this, a large amount of CNT is required to fabricate wirings. This implies that most CNTs in the composite are not utilized. The TF method requires a high-power laser to ablate the polymer.

From the viewpoint of material sustainability, the efficient utilization of CNTs is expected such as the recycling of unused donors in LIFT method and the unused embedded-CNTs in TF method. However, to the best of our knowledge, there have been no reports on recycling unused CNTs in both LIFT and TF methods.

In this study, to solve the issues mentioned above, a novel fabrication method of CNT wirings, which is based on the LIFT and TF method, was developed. Using this method, CNT wirings can be directly fabricated on PP films using low-cost semiconductor laser at room temperature (RT) under atmospheric pressure. The resistance of the CNT wirings varied from 0.789 to 114 kΩ/cm as the laser conditions varied. It is possible to form alternating high- and low-resistance regions in a single wire. The formation mechanism of CNT wiring with tunable resistance was discussed from both experimental results, such as microscopic structural observation using cross-sectional scanning electron microscopy (XSEM) and microscopic Raman imaging, and simulation results, such as heat conduction in the film during local laser heating. The recovery of unused CNTs and their reuse as source materials for CNT wiring is also demonstrated in this study.

## Method

### Material preparation

**Figure 1:**
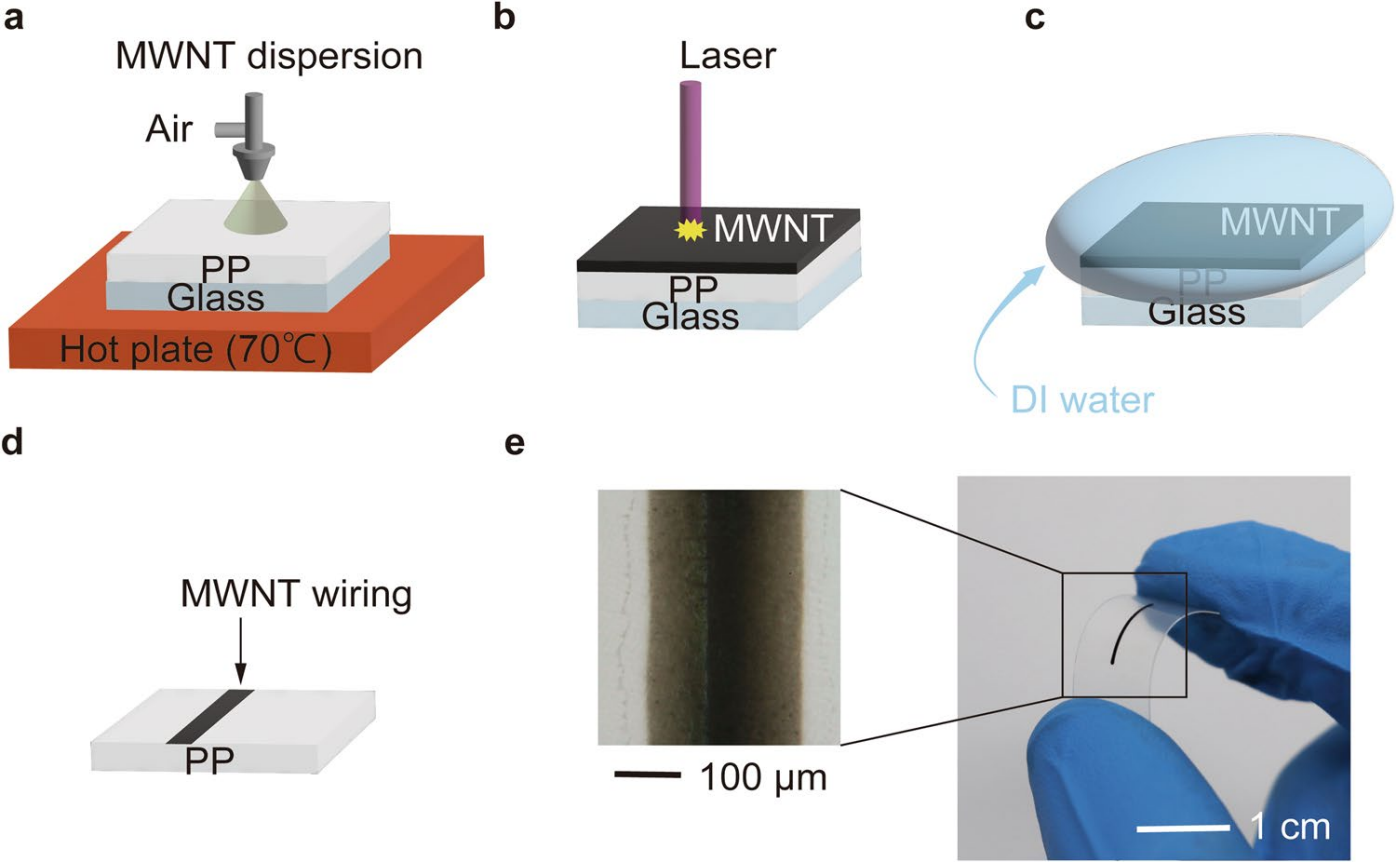
Schematic illustration of fabrication of CNT wiring on polypropylene substrate. (**a**) Schematic of CNT film formation on PP. (**b**) Laser irradiation of CNT film on PP. (**c**) Schematic of cleaning with DI water after laser irradiation and (**d**) after cleaning. (**e**) Fabricated CNT wiring on PP under bending.

The proposed fabrication method is shown schematically in Fig. 1. Multi-walled carbon nanotubes (MWNTs) dispersed in water (2.0 wt%) were purchased from Meijo Nano Carbon Co., Ltd., Japan (MW-I). To prepare the solution for spray deposition, the MWNT dispersion was diluted by mixing 12 mL of the as-received solution with 20 mL of deionized (DI) water. The dispersion was then poured into the solution reservoir of a custom-made spraying machine^23^. PP films [thickness 200 µm; size 5 × 5 cm; P466-1, (MISUMI Corporation, Japan)] were fixed on the surface of a hot plate (HP-1SA, AS ONE Corporation, Osaka, Japan) and heated in air at 70 °C. The CNT dispersion was sprayed onto the heated PP films. The spray area was 120 × 80 mm, and two PP films were placed in this area (Fig. 1a). The mean thickness of the MWNT films was approximately 10 µm. Figure 1b shows the MWNT film on the PP film located on a motorized X–Y stage (SGSP20-35, SIGMAKOKI CO., LTD., Japan). This was irradiated with a laser (DL-5146-101S, SANYO Electric Co., Ltd., Japan) (30–66 mW, λ = 405 nm) at different scanning speeds in the range of 5 µm/s–1 mm/s (the system schematics are also shown in Fig. S1). The laser was connected to a head with a built-in temperature controller (ALTH-103BC, ASAHIDATA SYSTEMS Co., LTD., Japan), and a collimator (ACH-10B, ASAHIDATA SYSTEMS Co. LTD., Japan) was equipped with a lens with a focal length of 4 mm and a working distance of 2.3 mm. The laser was driven by a laser driver (ALP-7033CC, ASAHIDATA SYSTEMS Co., Ltd., Japan). The films were then sonicated (1510J-MT, Yamato Scientific Co., Ltd., Japan) for 15 min to remove the remaining MWNT film and then blown with N_2_ (Fig. 1c,d). Figure 1e shows a photograph of a typical MWNT wiring fabricated using this method. All experiments were performed under ambient pressure at RT.

### Characterization

To measure the electrical transport properties, Ag paste (DOTITE D-500, FUJIKURA KASEI Co., Ltd.) was used to obtain ohmic contact between the MWNT wirings and probes. A source meter (2612A, Keithley, OH, USA) and probe station were used for current–voltage (*I*–*V*) measurements. The microstructure was observed using field-emission SEM (SUPRA 40, Carl Zeiss, Jena, Germany). The width of the MWNT wiring was characterized by optical microscopy (HISOMET2, Union Optical Co., LTD., Japan). The resistance under bending was measured with bending radii varying from 4.8 to 16 mm. To show that resistance can be controlled as a function of laser scanning speed, the temperature distribution was measured using thermography (IRC30, Teledyne FLIR LLC). A voltage of 32 V was applied using a DC power supply (GPD-2303S, Good Will Instrument Co., Ltd., Taiwan). The Raman spectra was obtained using Raman microscope (inVia Reflex, Renishaw plc., UK).

### Simulation methods

Heat conduction simulations based on the finite element method (FEM) were performed using COMSOL Multiphysics software. The model structure for the FEM simulation was a multilayer consisting of a CNT film with a thickness of 20 µm and a PP film with a thickness of 200 µm. The thermal conductivity of the CNT film and PP film were 1.2 and 0.2 W/mK, respectively. The laser was converted to heat in the CNT film and the heat was applied to the CNT and PP film. In this simulation, instead of a laser, Gaussian heat flux was applied to the CNT film. The standard deviation of the Gaussian distribution was one-third of the spot diameter (1340 μm).

A Joule heat simulation based on FEM was also performed using COMSOL Multiphysics. Fig. S2 shows the simulated device structure consisting of the PP films (height 16 mm, width 65 mm) and MWNT wirings. MWNT wiring with resistances of 1 and 20 kΩ/cm in a single wire was fabricated by adjusting the width of the wiring.

### Recycling

In this method, MWNTs on PP film that has not been irradiated by the laser may be wasted. From the viewpoint of material sustainability, it is important to recycle the unused MWNTs. We performed a demonstration of the recycling through following procedure. First, a MWNT film was prepared on a PP film using the procedure described above. Second, the MWNT wirings were fabricated by laser irradiation with an irradiation power of 66 mW and a scan speed of 1 mm/s. Third, after the laser-irradiated film was sonicated in 50 mL of DI water for 15 min, the MWNT wirings on the PP film and an MWNT aqueous solution were obtained. The obtained solution was sonicated using an ultrasonic homogenizer (FS300N, Shenzhen XinzhiBang Inst & Eq. Co., Ltd., China) at an output power of 300 W for 10 min. Fourth, the *I*–*V* properties of the wirings were measured. Finally, once this process was complete, we returned to the second step of this procedure and fabricated new MWNT wirings on a new PP film using the recycled MWNT solution. The recycling procedure was repeated four times, and we obtained the resistances of MWNT wirings as a function of the number of recycling.

## Result and discussion

**Figure 2:**
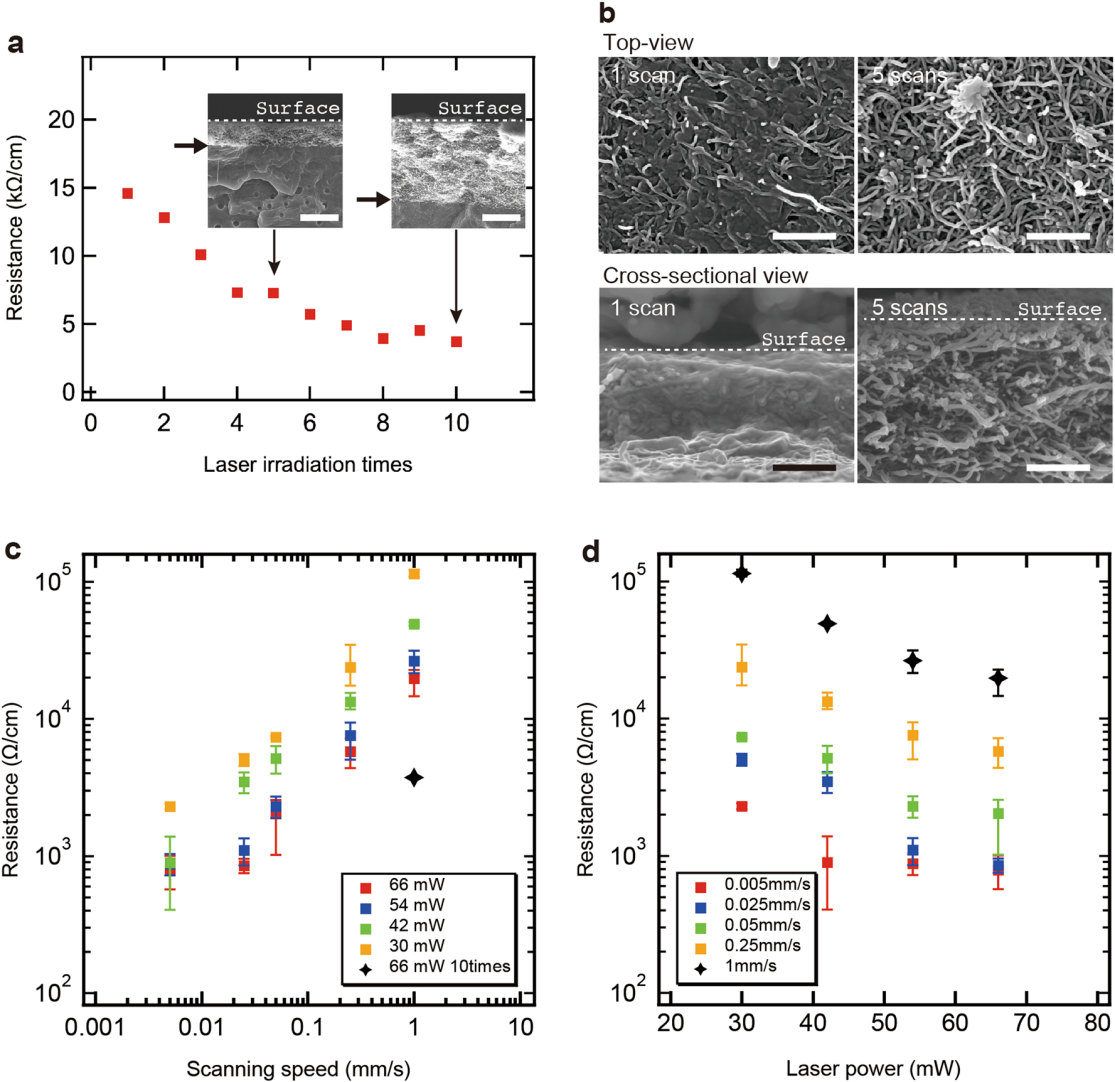
(**a**) Resistance per cm as a function of laser irradiation time (laser power 66 mW and scanning speed 1 mm/s). The insets show the cross-sectional SEM images of the samples after 5 and 10 scans. Scale bars are 2 μm. (**b**) Top-view and cross-sectional SEM images. Scale bars are 0.5 μm. Resistance per cm as functions of (**c**) scanning speed and (**d**) laser power. Error bars indicate maximum and minimum values.

Figure 2a shows the electrical resistance as a function of the number of laser scans, with a laser power of 66 mW and a scanning speed of 1 mm/s. The samples were not sonicated between laser scans. In other words, the samples were sonicated only once at the end of the multiple laser scans. After a single laser scan, the resistance was 14.6 kΩ/cm. The resistance of the area without wiring was above 40 GΩ, which is the measurement limit, so MWNTs only existed in the area irradiated by the laser. There were no MWNTs in other areas. The resistance decreased with an increasing number of scans. The resistance was 14.6 kΩ/cm per 1 scan and decreased to 3.72 kΩ/cm after 10 scans. Over 7 scans, the resistances were similar for each value. The XSEM images around the MWNT wirings are shown in the insets of Fig. 2a. It was found that there was a boundary plane where the contrast clearly changed (shown by the arrows). Differences, in contrast, may reflect differences in the concentrations of MWNTs. The thickness of the bright region, where the concentration of MWNTs could be high, was approximately 1.4 μm after 5 scans and approximately 4.8 μm after 10 scans, indicating that the thickness increases with the number of scans.

To investigate the local structure of the bright region in more detail, the SEM images were observed. Figure 2b shows the top-view SEM images and magnified XSEM images of the MWNT wirings after one and five scans. After one scan, fibrous nanostructures embedded in the film were observed in the bright region, indicating that MWNTs and PP were mixed in the film. However, after five scans, the fibrous nanostructures, which appeared to be MWNTs, were found to be entangled. It appears that the relative PP concentration for the five scans was lower than that for one scan.

Figure 2c shows the average electrical resistance as a function of the scanning speed at various laser powers. For each irradiation condition, a couple of samples were prepared, and their *I*–*V* properties were measured. The resistance decreased with increasing scanning speed, and the distribution followed an approximate power-law distribution. The MWNT wiring exhibited a controllable resistance from 0.789 to 114 kΩ/cm. Figure 2d shows the average electrical resistance as a function of laser power at various scanning speeds. The resistance decreased exponentially with the laser power. Samples prepared under the same irradiation condition were found to exhibit resistances of the same order of magnitude.

**Figure 3:**
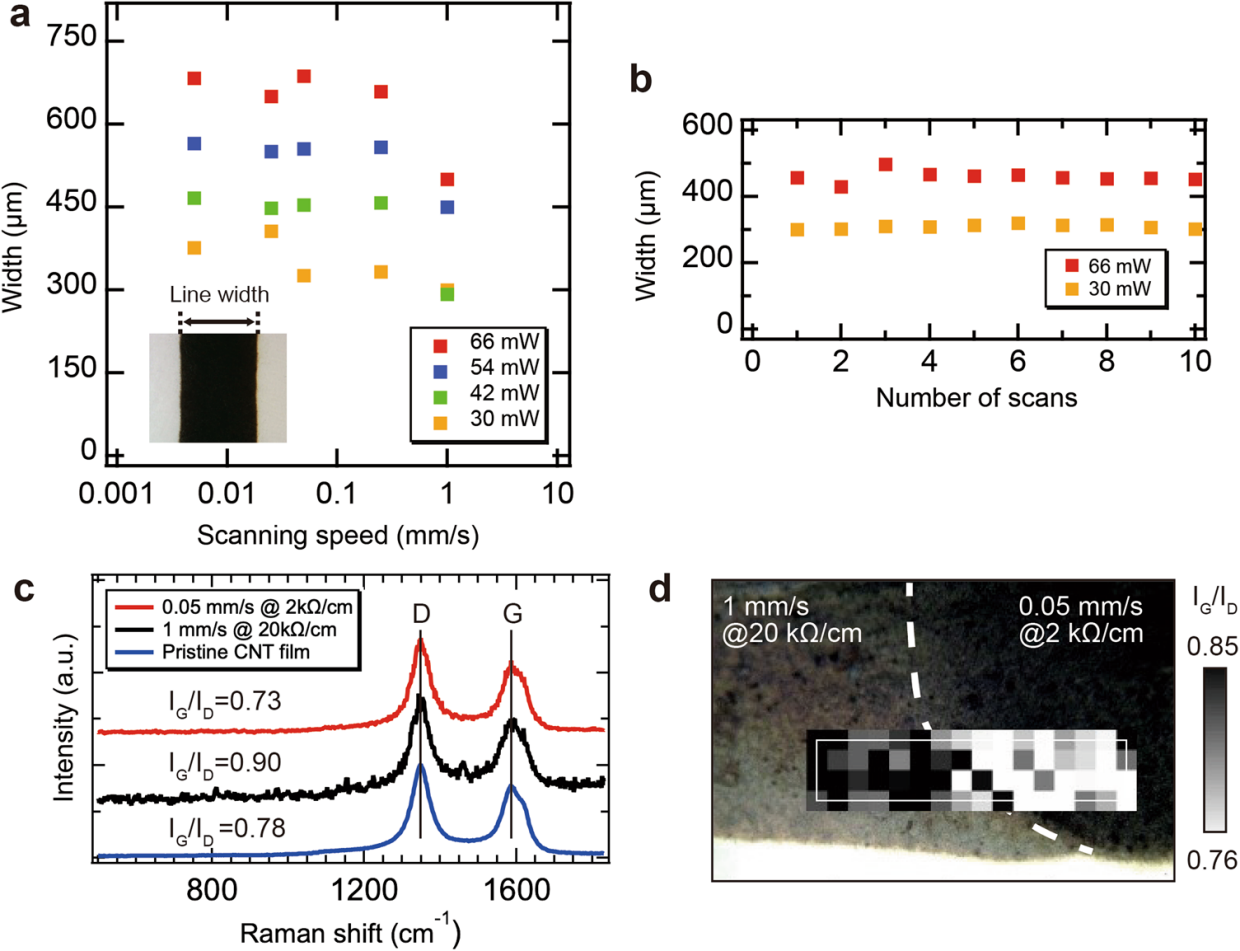
(**a**) Line width of MWNT wirings as a function of the laser scanning speed for various laser powers. (**b**) Line width of MWNT wirings as a function of the number of scans (scanning speed 1 mm/s). (**c**) Raman spectra of MWNT wirings for various laser scanning speeds. (**d**) Raman mapping of G–D ratio of MWNT wirings for various scanning speeds with different resistance values within a single wire.

Next, the average linewidth of the MWNT wiring was measured using an optical microscope. The linewidth was defined as the length of the black area perpendicular to the laser scanning direction (see inset, Fig. 3a). Samples were prepared and measured three times. Figure 3a shows the average linewidths as a function of the laser scanning speed for various laser power settings. The linewidths rarely changed with the scanning speed of the laser, except at a scanning speed of 1 mm/s. In contrast, the linewidths increased with the laser power and varied from 292 to 683 µm depending on the laser conditions. Figure 3b shows the linewidth as a function of the number of laser scans for laser powers of 30 and 66 mW at a scanning speed of 1 mm/s. The linewidths rarely changed with the number of scans, and the widths increased with the laser power.

Raman analysis was performed to investigate the effect of laser irradiation on the crystallinity of the MWNTs. Figure 3c shows the Raman spectra of the MWNT wirings at various laser scanning speeds and the MWNT film before the laser irradiation. There were two characteristic peaks^24,25^: the D band (~ 1350 cm^−1^) and the G band (~ 1580 cm^−1^), which represent the defects and graphitic nature of *sp*^2^ bonds, respectively. The G/D ratio indicates the crystallinity of the MWNTs. These were estimated to be 0.73 for a scanning speed of 0.05 mm/s, 0.90 for a scanning speed of 1 mm/s, and 0.78 for pristine MWNTs. Therefore, the higher the scanning speed, the higher the crystallinity of the MWNT wirings. Compared with the crystallinity of the pristine MWNTs, at slower scanning speeds, the crystallinity was found to be slightly degraded. This trend suggests that, depending on the different irradiation conditions, the laser local heating causes either improvement of the crystallinity or formation of defects.

Figure 3d shows a superimposed optical microscope image and Raman mapping at the interface of the samples fabricated at different scanning speeds in a single wiring. It was confirmed that the G/D ratio changed significantly at the interface where the scanning speed was switched. MWNT wirings with slower scanning speeds had smaller G/D ratios, indicating low crystallinity. We believe that the MWNTs were oxidized by increasing the surface temperature of the MWNT for long-time laser irradiation, which may explain the relationship between the lower scanning speed and lower crystallinity. Although MWNTs with more defects should have higher resistance, this result showed the opposite trend. The reasons for this were considered as follows.

The resistance per unit length (*R*) can be expressed as $R=\frac{\rho }{wd}$, according to Ohm's law^26^, where *ρ*, *w*, and *d* are the resistivity, width, and depth of the wiring, respectively. The linewidths were independent of the scanning speed and number of laser scans, as shown in Fig. 3a and b. On the other hand, as shown in Fig. 2a and b, both the thickness of the wiring and the relative concentration of MWNTs (i.e., corresponding to *ρ* in the wiring) were increased with increasing the number of irradiations. If the temporal accumulation of photon energy determines the thermal fusion of MWNTs and PP films, an increase in the number of scans might be synonymous with a decrease in the scanning speed. Therefore, the resistance of the wiring is considered to be mainly determined by *ρ* and *d*. The reason for the decrease in the resistance of wiring despite the degradation of local crystallinity during slow scan might be that both the reduction of *ρ* and the increase of *d* were more dominant than the degradation of local crystallinity of MWNTs.

**Figure 4:**
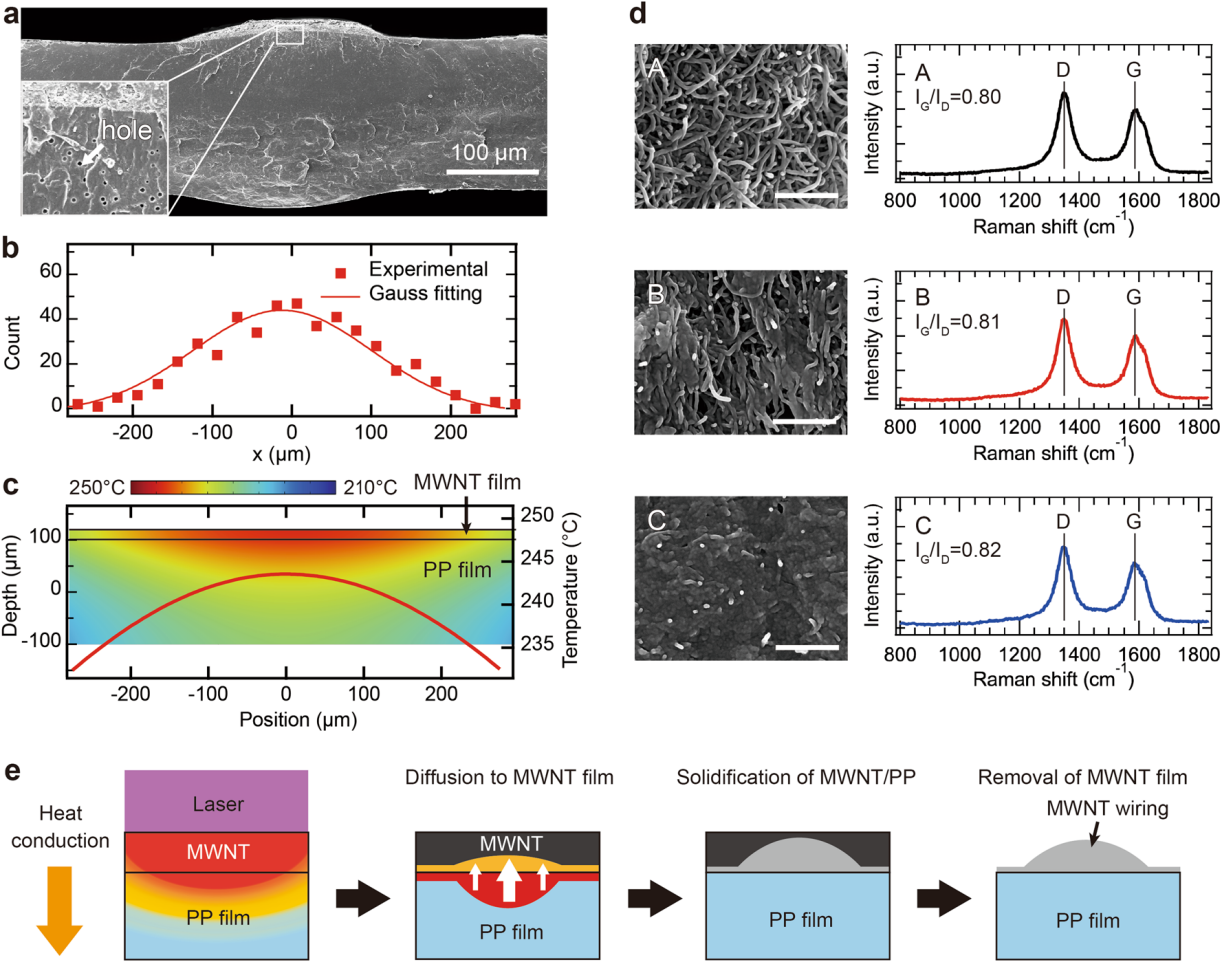
(**a**) XSEM images of MWNT wiring (laser power 66 mW, scanning speed 0.05 mm/s). (**b**) Hole density distribution in PP. (**c**) Temperature distribution in PP simulated with COMSOL Multiphysics. (**d**) Top-view SEM images and Raman spectra of the MWNT wiring with a laser power of 66 mW and a scanning speed of 0.05 mm/s at center to edge in the wiring. A, B, and C indicate the center, middle, and edge of the MWNT wiring, respectively. Scale bars are 0.5 μm. (**e**) Speculated formation mechanism of MWNT wirings.

To investigate the formation mechanism of the MWNT wirings, XSEM observations and heat conduction simulations based on FEM were performed. Figure 4a shows an XSEM image of the MWNT wiring with a laser power of 66 mW and a scanning speed of 0.05 mm/s. All areas in Fig. 4a are black when observed from above. A thick layer, which appeared to be a MWNT film, was found in the central area. Its length was over 200 µm. Submicron holes were observed between the MWNT film boundaries at a depth of 60 μm. To determine the hole distribution, the density distribution of the holes in a region with a width of 25 μm and an average depth of 60 μm was obtained from the XSEM images. Figure 4b shows the measured density distribution of the holes and the Gaussian function fitting. The full width at half maximum of the Gaussian function is 264.74 µm, and the distribution of holes is consistent with the thick MWNT layer.

It has been reported that holes are formed when plastic freestanding films are heated, and the number of holes increases with the temperature^27^. Therefore, the thick MWNT layer was considered to be a high-temperature region. The temperature distribution of the film under laser irradiation was investigated using the FEM. Figure 4c shows a superimposed temperature distribution at the boundary of MWNT layer and PP and temperature distribution of the film. Heat is preferentially conducted in the horizontal direction rather than the vertical direction because the thermal conductivity is higher for MWNT films than for PP. The temperature around the surface of the ensemble was higher than that in the film. This is consistent with the distribution of the number of holes, indicating that the region with a thick MWNT layer is a high-temperature region.

Figure 4d shows the top-view SEM images and Raman spectra of the MWNT wiring, of which the irradiation conditions were the laser power of 66 mW and the scan speed of 0.05 mm/s, at the center (A), middle (B), and edge (C) positions of the wiring. The distance between each position was approximately 100 μm. Although MWNTs were observed clearly in the center region, at the middle region, a structure in which some MWNTs were buried in the PP film was observed. At the edge region, many MWNTs were embedded in the PP film. On the other hand, the G/D ratios of the Raman spectra were almost constant within the observed regions.

PP diffuses widely into the MWNT film because the high-temperature PP has a high diffusion coefficient^28^. Consequently, around the center region, the thickness of the MWNT–PP composite layer was increased, as shown in Fig. 4a. Moreover, a large amount of PP was assumed to be evaporated, resulting in exposure of the MWNTs. Meanwhile, since the temperature decreases in the region near the edge, the thickness of fusion region is considered to be thinner due to the lower diffusion coefficient of PP. The buried film of MWNTs in the PP was formed because the amount of evaporated PP was decreased at a lower temperature. In this case, the temperature difference at different locations rarely affected the crystallinity of the MWNTs. However, it affected the diffusion and evaporation of PP.

In summary, the formation mechanism of MWNT wirings can be considered as follows. As shown in Fig. 4e, the MWNT film is irradiated by a laser and generates heat because the MWNTs have a high photothermal conversion efficiency^29^. The thermal conductivities of the MWNT film and PP were 15 and 0.180 W/mK, respectively^30,31^. Thus, heat preferentially conducts in the horizontal direction, resulting in high temperatures at the MWNT–PP interface and low temperatures in the PP films. The high-temperature PP diffused into the MWNT film. At the center of the laser, which is the high-temperature region, a large amount of PP diffuses into the MWNT film. However, at the edge of the laser, where the temperature is lower, a small amount of PP diffuses into the MWNT film. The diffused PP in the MWNT film forms a PP/MWNT composite layer. Thus, a thick PP/MWNT composite was formed at the center of the laser, whereas a thin PP/MWNT composite was formed at the edge of the laser. As the laser power increased, the thickness of the PP/MWNT composite increased, which may have decreased the resistance because of the large number of MWNTs in the thick MWNT layer.

**Figure 5:**
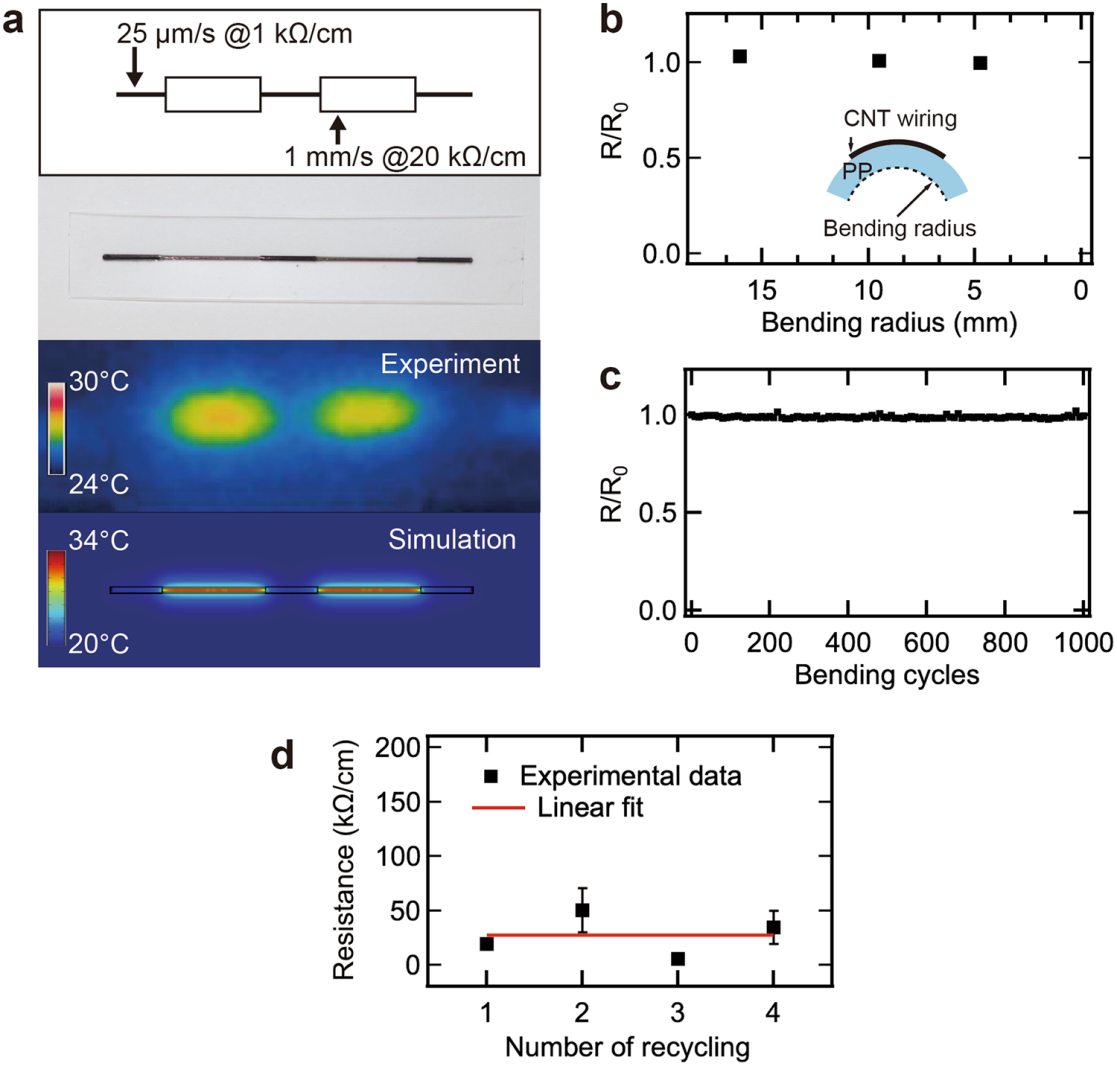
(**a**) Temperature map of MWNT wirings and simulation result with photograph and schematic diagram. (**b**) *R*/*R*_0_ values with different bending radii. *R*_0_ is the initial resistance. (**c**) Repeated bending test with a bending radius of 9.5 mm. (**d**) Resistance per cm as a function of number of recycling.

We found that the resistance of MWNT wiring can be changed by controlling the laser conditions. To visualize the change in resistance in the wiring, a voltage was applied to one wiring at a varying scan speed, and the temperature distribution was measured using thermography. Figure 5a shows the schematic, photograph, thermographic image, and simulation image. The temperature increases because of Joule heating in the region where the laser scanning speed is fast. This result is consistent with the simulation results. It was shown that wires with different resistance values can be formed by simply changing the laser scanning speed.

Figure 5b shows the ratio of the resistance (*R*) of the MWNT wiring under bending and the resistance (*R*_0_) under flat conditions as a function of the bending radius. The resistance of the MWNT wiring remained constant under bending conditions. To investigate the reliability of the MWNT wiring, a repeated bending test was performed. The film was bent 1000 times with a radius of curvature of 9.5 mm. As shown in Fig. 5c, the resistance of the MWNT wiring remained constant even after 1000 bending cycles, indicating that the wiring is highly reliable without structural degradation due to bending. The fabricated MWNT wiring exhibited flexibility. MWNT and polymer composites have been reported to vary in resistance under bending because the polymer matrix is stretched, increasing the MWNT–MWNT distance and decreasing the number of conductive paths, resulting in a decrease in resistance^32–34^. The constant resistance of the MWNT wirings under bending can be attributed to the high density of MWNTs in the MWNT–PP composite layer. As shown in Fig. 2b, the PP/MWNT composite layer formed a dense and random MWNT film. When the MWNT wiring was bent, the matrix was stretched, while the number of conductive paths did not change because of the significant area of contact between the MWNT and MWNT–MWNTs. Therefore, it was concluded that the resistance of the wiring did not change under bending.

Next, we demonstrated the recycling of unused MWNTs on PP films. We made MWNT aqueous solutions from the unused MWNTs, which are on the area not irradiated by the laser. The recovered MWNT solution was used for spraying again. Figure 5d shows the resistance of the MWNTs as function of number of recycling. The resistance of the MWNT wiring fabricated using the outlined method remained almost constant up to a factor of four. It was demonstrated that this method can reduce the number of MWNTs used and can use them more efficiently than conventional thermal fusion methods.

## Conclusion

In this study, the formation of MWNT wirings was demonstrated by spray-depositing MWNTs on a PP film and irradiating them with a laser. The fabricated MWNT wirings exhibited controllable resistance ranging from 0.789 to 114 kΩ/cm, depending on the laser conditions. The linewidth was found to depend not on the laser scanning speed, but on the laser intensity. XSEM observation of the MWNT wiring revealed that a thick MWNT layer was formed in the center of the MWNT wiring. In addition, the formation of a hole under the thick MWNT layer and the simulation results indicate that the thick MWNT layer was a high-temperature region. Therefore, it was concluded that the diffusion of high-temperature PP into the MWNT film formed a thick MWNT layer, resulting in a decrease in resistance.

The MWNT wirings fabricated using the method outlined showed no change in resistance under bending. The MWNTs that were not used for wiring could be easily recycled, and the resistance did not change after recycling. The fabricated MWNT wirings are flexible, require low MWNT usage, and can be fabricated directly on PP with controllable resistance under a non-vacuum atmosphere. This technology could be used to fabricate carbon wirings and carbon devices for flexible sensors, which are expected to become popular on large scale markets.

## Supplementary Information

Supplementary Figures.

## Data Availability

The datasets used and/or analyzed during the current study available from the corresponding author on reasonable request.
