# Supplementary material for: Direct formation of carbon nanotube wiring with controlled electrical resistance on plastic films
Source: Sci Rep. 2023 Feb 8;13:2254. doi: 10.1038/s41598-023-29578-w (PMC9908902; doi:10.1038/s41598-023-29578-w)
Supplement: Supplementary file 1 — Supplementary Figures. [file 41598_2023_29578_MOESM1_ESM.docx]

**Direct Formation of Carbon Nanotube Wiring with Controlled Electrical Resistance on Plastic Films**

To be submitted to ***Scientific Reports***

**Hiroaki Komatsu, Takahiro Matsunami, Yosuke Sugita, and Takashi Ikuno***

Department of Applied Electronics, Graduate School of Advanced Engineering, Tokyo University of Science, Katsushika, Tokyo 125-8585, Japan

*****tikuno@rs.tus.ac.jp

**Supplemental information**


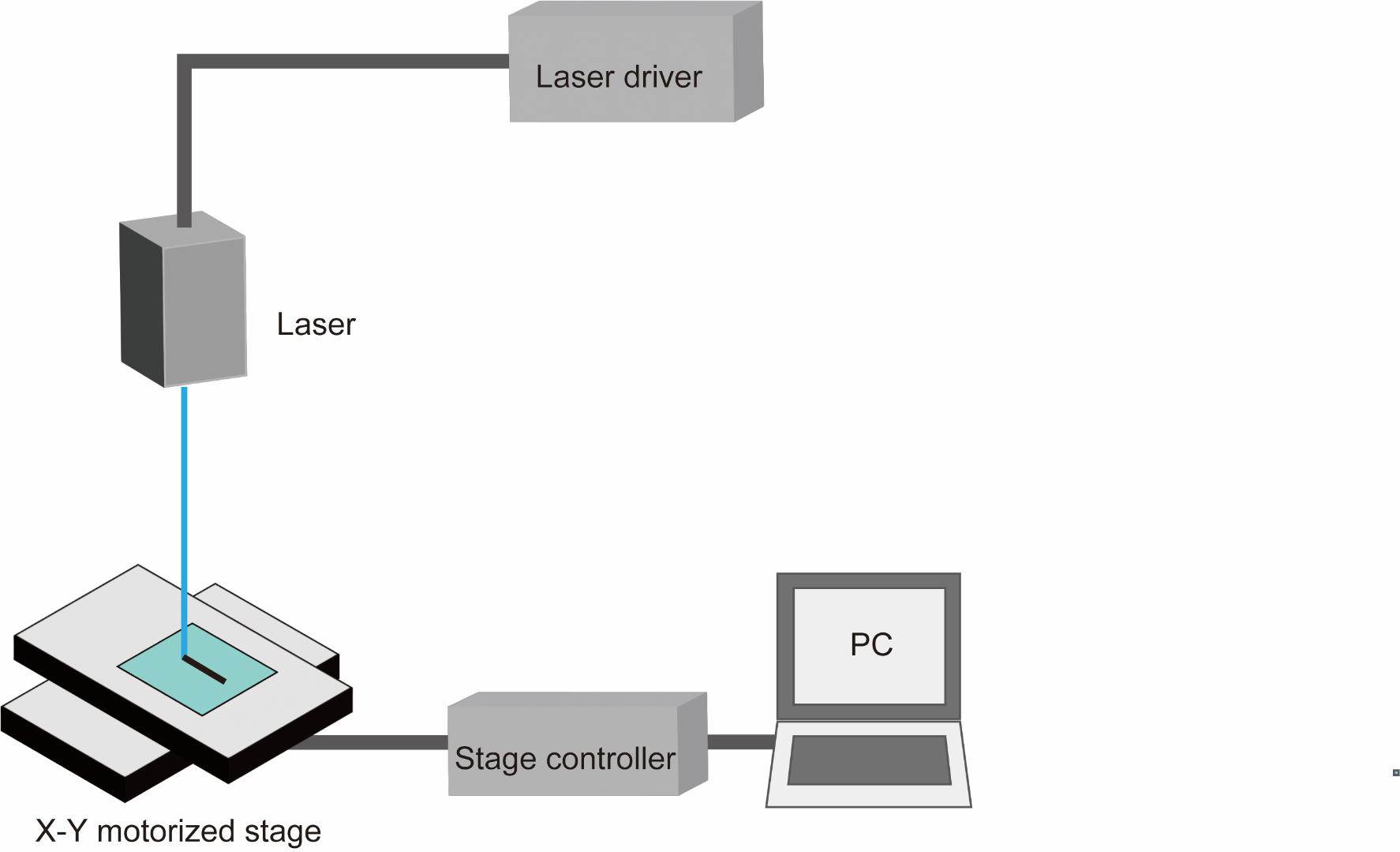


**Figure S1.** Schematics diagram of laser irradiation system


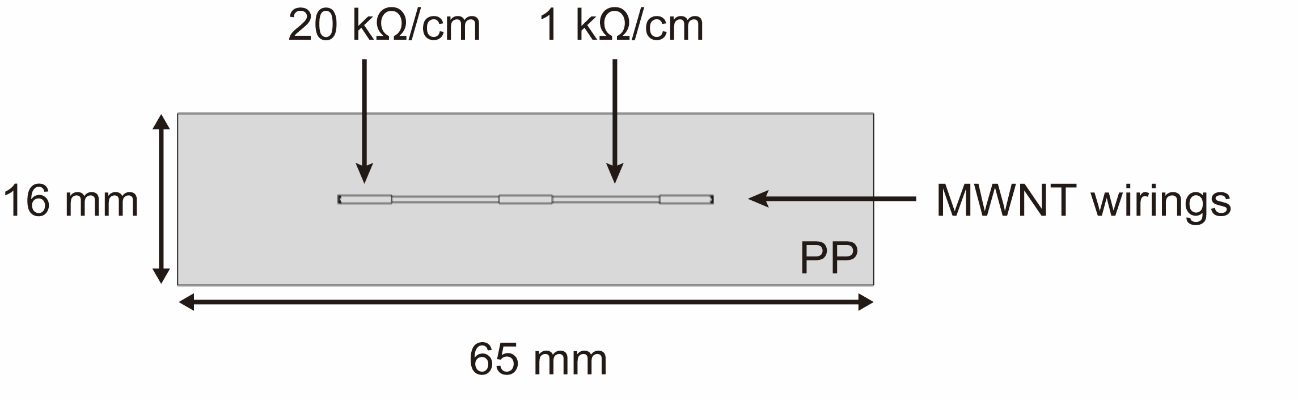


**Figure S2.** Simulated device structure consisting of the PP films
